# Retrospective Evaluation of the Incidence, Clinical Characteristics and Outcomes of Gram-Negative Bacterial Infections in Patients with Hematologic Malignancies

**DOI:** 10.3390/pathogens14121238

**Published:** 2025-12-04

**Authors:** Firdevs Aksoy, Hanife Nur Karakoc Parlayan, Munire Agirman, Esra Ozkaya, Mehmet Sonmez, Gurdal Yilmaz

**Affiliations:** 1 Department of Infectious Disease and Clinical Microbiology, Faculty of Medicine, Karadeniz Technical University, Trabzon 61080, Türkiye; faslanaksoy@ktu.edu.tr (F.A.); gurdalyilmaz@ktu.edu.tr (G.Y.); 2 Department of Medical Microbiology, Faculty of Medicine, Karadeniz Technical University, Trabzon 61080, Türkiye; esraozkaya@ktu.edu.tr; 3 Division of Hematology, Department of Internal Medicine, Faculty of Medicine, Karadeniz Technical University, Trabzon 61080, Türkiye

**Keywords:** bloodstream infections, hematologic malignancies, Gram-negative bacteremia, mortality

## Abstract

Background: Patients with hematologic malignancies are highly vulnerable to Gram-negative bacterial bloodstream infections (GNB-BSIs) due to underlying disease-related immunosuppression, intensive chemotherapy, and repeated invasive interventions, rendering these infections a significant cause of morbidity and mortality in this population. This study aimed to evaluate the epidemiological, clinical, and microbiological features of GNB-BSIs in hospitalized patients with hematologic malignancies, and to compare clinical and microbiological factors between survivors and non-survivors. Methods: We conducted a retrospective cohort study in a tertiary university hospital hematology ward in Türkiye, including adult patients diagnosed with BSIs due to Gram-negative bacteria between January 2005 and December 2024. Demographic characteristics, microbiological profiles, antimicrobial resistance rates, and clinical outcomes were analyzed. We compared survivors and non-survivors to determine differences in clinical and microbiological characteristics. Results: A total of 321 patients with hematologic malignancies experienced 441 episodes of GNB-BSIs. The median age was 46 years, and 59% of them were male. The most frequently isolated pathogen was *Escherichia coli* (53.3%), followed by *Klebsiella* spp. (20.6%) and *Pseudomonas* spp. (7.5%). Extended-spectrum β-lactamase-producing/third-generation cephalosporin-resistant (ESBL/3GCR) and carbapenem-resistant isolates were observed in 21.1% and 13.3% of isolates, respectively. The overall mortality rate was 26.5%. ICU admission, multidrug resistance, and persistent bacteremia were observed more often among non-survivors. Additionally, prolonged fever duration (median 8 vs. 3 days, *p* < 0.0001), elevated CRP (*p* = 0.001), and higher procalcitonin levels (*p* = 0.046) were detected in non-survivors. Conclusions: In patients with hematologic malignancies, *E. coli* and *Klebsiella* spp. remain the predominant pathogens causing bloodstream infections, while persistent bacteremia, ESBL/3GCR, and carbapenem resistance are associated with higher mortality. Notably, carbapenem resistance showed a temporal increase over the study period, underscoring the need for continuous surveillance and timely adaptation of empirical treatment strategies.

## 1. Introduction

Individuals with hematological malignancies (HM) face a heightened risk of infectious complications due to underlying immune dysfunction and the intensive therapies they receive, such as chemotherapy, radiotherapy, and invasive procedures. The patients undergoing cancer treatment often have weakened immune defenses as a result of the disease and its therapies, leaving them highly prone to infectious complications [1,2,3]. Bloodstream infections (BSIs) rank among the most life-threatening infections in this patient population, often dictating clinical outcomes. BSIs therefore represent not only an important driver of morbidity and mortality but also a significant barrier to the successful administration of cancer-directed therapies [4,5].

The epidemiology of bacterial infections in this group has undergone substantial changes in recent decades, with numerous studies reporting a rising incidence of BSIs caused by resistant pathogens, particularly Gram-negative bacteria (GNB) [6,7,8]. The emergence of antimicrobial resistance has compounded the clinical challenge. Multidrug-resistant GNB, such as extended-spectrum β-lactamase (ESBL) producing and carbapenem-resistant strains, have been increasingly reported in this population. These resistant organisms are strongly associated with adverse outcomes and higher mortality, underscoring the need for timely and effective empiric therapy [4,9]. It has made the choice of empiric antibiotic therapy considerably more challenging. Selecting optimal empirical antibiotics has become increasingly complex, posing a major challenge to improving patient outcomes [10,11].

A thorough understanding of the epidemiology of resistant bacterial BSIs is essential to inform empirical antibiotic strategies for high-risk patients [12]. Such knowledge enables physicians to tailor treatment regimens, avoid inappropriate empirical coverage, and implement antimicrobial stewardship measures while ensuring adequate initial therapy. Beyond immediate clinical outcomes, infections in HM patients frequently complicate treatment courses, leading to delays in chemotherapy or transplantation, prolonged hospitalizations, increased healthcare costs, and ultimately poorer survival [10].

Given these considerations, continuous surveillance of bloodstream infections in patients with hematological malignancies is crucial to guide evidence-based interventions. Understanding local epidemiology is particularly important in our setting, where regional differences in antimicrobial resistance patterns and center-specific treatment practices may influence the clinical course and outcomes of Gram-negative bacterial bloodstream infections (GNB-BSIs). However, long-term local data on the epidemiology, resistance patterns, and clinical outcomes of GNB-BSIs in hematology patients remain limited. In this study, we aimed to evaluate epidemiological, clinical, and microbiological characteristics of GNB-BSIs in hospitalized patients with hematologic malignancies. Moreover, we assessed temporal trends in antimicrobial resistance and examined clinical and microbiological factors associated with mortality.

## 2. Materials and Methods

### 2.1. Study Design and Setting

This retrospective observational study was conducted at Karadeniz Technical University, a tertiary care center, and included HM patients with bacteremia. Hospitalized patients who were followed between January 2005 and December 2024 were retrospectively enrolled. Given the long study period, temporal changes in diagnostic criteria, laboratory methods, and susceptibility testing systems were taken into account during data review.

### 2.2. Data Collection

Patients’ demographic and clinical data, including age, sex, underlying hematologic malignancy, length of hospital stay, ICU admission, duration of neutropenia, antibiotic regimen, and clinical outcome (survival status), along with relevant laboratory parameters such as the isolated Gram-negative organism, antimicrobial resistance profile, C-reactive protein (CRP), procalcitonin, white blood cell count, and neutrophil count, were retrospectively obtained from the Infection Control Committee records, consultation forms of the Department of Infectious Diseases and Clinical Microbiology, and the Hospital Information System.

All antibiotics administered for either treatment or prophylactic purposes were considered. The recorded antibiotic regimens included β-lactams, quinolones, carbapenems, glycopeptides, and their combinations. Isolated microorganisms were classified as either fermentative or non-fermentative Gram-negative bacteria.

Institutional antimicrobial prophylaxis protocols and laboratory reference ranges were applied according to the standards in use at the time of patient management, while antimicrobial susceptibility results were retrospectively interpreted in accordance with the current CLSI/EUCAST guidelines to ensure consistency across the study period.

The primary endpoint of the study was the distribution and frequency of GNB isolated from blood cultures of patients with hematological malignancies, whereas mortality and ICU admission were prespecified as secondary clinical outcomes.

### 2.3. Definitions

Upon presentation with fever and/or clinical suspicion of infection, empiric broad-spectrum intravenous antibiotics were initiated in accordance with the guidelines [13,14]. The attending physicians selected the antibiotic treatment for the patients. The empirical therapy of neutropenic fever consisted of cefepime 2 g/8 h, ceftazidime 2 g/8 h, or piperacillin/tazobactam 4 g/0.5 g/6 h I.V. Antimicrobial resistance trends were evaluated across the study period to assess their impact on empirical antibiotic protocols. In response to rising resistance rates to ESBL/3GCR GNB, institutional guidelines were progressively updated to favor broader-spectrum agents, such as piperacillin/tazobactam and carbapenems, in high-risk patients. In the later years, the emergence of carbapenem-resistant isolates prompted the selective use of combination regimens and the adoption of susceptibility-guided therapies when clinically indicated.

Antimicrobial prophylaxis (AP) is recommended to prevent primary or secondary infections and to limit microbial colonization; however, it should be employed only for well-established indications. Inappropriate use may lead to increased treatment costs, toxicity, and the emergence of antimicrobial resistance [15]. In our institution, antibiotic prophylaxis, such as quinolones, is not routinely administered to HM patients. Trimethoprim/sulfamethoxazole (160/800 mg) was administered three times weekly to prevent *Pneumocystis jirovecii* infection, and posaconazole was used as antifungal prophylaxis in patients with acute myeloid leukemia.

Fever is defined as an axillary temperature exceeding 38 °C on two occasions at least 1 h apart or 38.5 °C on one occasion. Neutropenia was defined as an absolute neutrophil count ≤500 cells/mm.

Bacteremia was defined by the detection of at least one positive blood culture. A follow-up blood culture was described as a repeat culture performed 48–72 h after the initial positive result was detected.

Persistent bacteremia was defined as the presence of positive blood cultures for the same organism for ≥48 h after the initiation of appropriate antimicrobial therapy.

Antibiotic resistance patterns, including those of extended-spectrum beta-lactamase-producing/third-generation cephalosporin-resistant (ESBL/3GCR) and carbapenem-resistant strains, were examined.

### 2.4. Study Population

All adult patients (≥18 years old) diagnosed with hematologic malignancies and admitted to the hospital between January 2005 and December 2024 were retrospectively screened. Patients who developed GNB-BSIs during hospitalization were included in the analysis. Inclusion criteria included a confirmed diagnosis of a hematologic malignancy, such as leukemia, lymphoma, or other disorders like multiple myeloma (MM) or myelodysplastic syndrome (MDS); at least one microbiologically confirmed GNB infection based on positive culture results; and complete clinical and microbiological data. For each patient, only the first positive blood culture was included in the analysis. Any subsequent positive cultures, obtained after documented culture negativity with at least a three-week interval, and/or those collected during subsequent hospitalizations, were considered separate episodes. Patients were excluded if they were under 18 years old, had no GNB infection, or had incomplete or missing microbiological records.

According to the underlying hematologic malignancy, patients were categorized into three groups: leukemia, lymphoma, and others. The leukemia group included patients diagnosed with acute lymphoblastic leukemia (ALL), acute myeloid leukemia (AML), chronic myeloid leukemia (CML), chronic lymphocytic leukemia (CLL), and other less common leukemia subtypes. The lymphoma group was classified into Hodgkin lymphoma and non-Hodgkin lymphoma.

### 2.5. Antimicrobial Testing

Blood cultures were processed using the automated culture system BACTEC 9240 (2005–2009, 2010–2014) (Becton and Dickinson Microbiology System, Franklin Lakes, NJ, USA), BACT/ALERT^®^ blood culture system (BioMérieux, Marcy-l’Étoile, France) (2003–2010), and BD BACTEC™ FX Blood Culture System (Becton and Dickinson Microbiology System, Franklin Lakes, NJ, USA) (2010–2024). The blood culture bottles were inoculated with the blood of patients following the manufacturer’s guidelines and were incubated until a positive signal was detected, with a 5-day incubation period. Upon observing a positive signal, the bottles were removed from the instrument, and Gram staining of the smears prepared from the positive blood culture bottles was performed, followed by streaking on agar plates. All of these plates were incubated at a temperature of 35–37 °C for a period ranging from 24 to 48 h.

The identification of bacterial isolates was performed using conventional methods, and BD Phoenix system (Becton Dickinson, Franklin Lakes, NJ, USA) (2005–2009, 2010–2014), Vitek-2 system (BioMérieux, Marcy-l’Étoile, France) (2009–2010), and MALDI-TOF mass spectrometer (Bruker Daltonic, Bremen, Germany) (2014–2024).

Antimicrobial susceptibility testing was performed with either BD Phoenix system (Becton Dickinson, Franklin Lakes, NJ, USA) (2005–2009, 2010–2024) and Vitek-2 system Vitek 2 (BioMérieux, Marcy-l’Étoile, France) (2009–2010). We used the current Clinical and Laboratory Standards Institute (CLSI) (2005–2015) and European Committee on Antimicrobial Susceptibility Testing (EUCAST) breakpoints (2015–2024) for each year to define susceptibility, intermediate, or resistance to these antimicrobial agents.

ESBLs were suspected by minimum inhibitory concentration (MIC) results and confirmed by double-disk synergy testing [16]. Carbapenemase-producing Enterobacterales were phenotypically detected by the modified Hodge Test or the modified carbapenem inactivation method (mCIM) [17].

Carbapenem-resistant GNB were defined as exhibiting non-susceptibility to at least one of three carbapenem antibiotics (imipenem, meropenem, and ertapenem). Stenotrophomonas maltophilia, which is intrinsically resistant to carbapenem antibiotics, was also classified as carbapenem-resistant GNB.

In calculating resistance rates, isolates without susceptibility testing for a specific antibiotic were excluded from the calculation of the denominator.

### 2.6. Statistical Analysis

Categorical variables were detailed as counts and percentages, whereas continuous variables were described as either means and standard deviations (SDs) or medians and interquartile ranges (Q1–Q3). Categorical variables were evaluated using the χ2 or two-tailed Fisher’s exact test. Odds ratios (ORs) and 95% confidence intervals (CIs) were calculated to evaluate the strength of any association that emerged, and either the Mann–Whitney U test or Student’s *t*-test, according to the variable distribution, was employed to compare the distribution of continuous variables. Survivor and non-survivor subgroups were compared to identify the differences between the two groups. Our research utilized IBM SPSS Statistics for Windows (Version 25.0, IBM Corp., Armonk, NY, USA). A *p* value of <0.05 was considered significant.

### 2.7. Ethical Considerations

The study protocol was approved by the Institutional Ethics Committee of Karadeniz Technical University (Approval No. 2025/169, Date: 7 July 2025). Patient confidentiality was maintained, and no identifiable data were used.

## 3. Result

A total of 321 cases, comprising 441 episodes of positive blood cultures, were included in the study. The mean age of the cohort was 46.2 ± 15.5 years, and 59% of them were male. Disease duration was less than one year in 159 episodes, one year in 119 episodes, between two and five years in 69 episodes, and longer than five years in 12 episodes. Notably, 88% of the episodes were neutropenic, and 98% presented with fever at the time of admission. The demographic and baseline clinical characteristics of the study population are summarized in Table 1.

**Figure 1:**
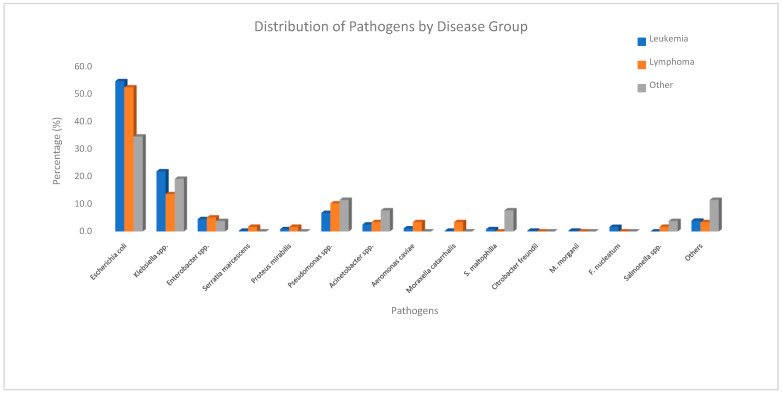
Distribution of pathogens by disease group.

**Figure 2:**
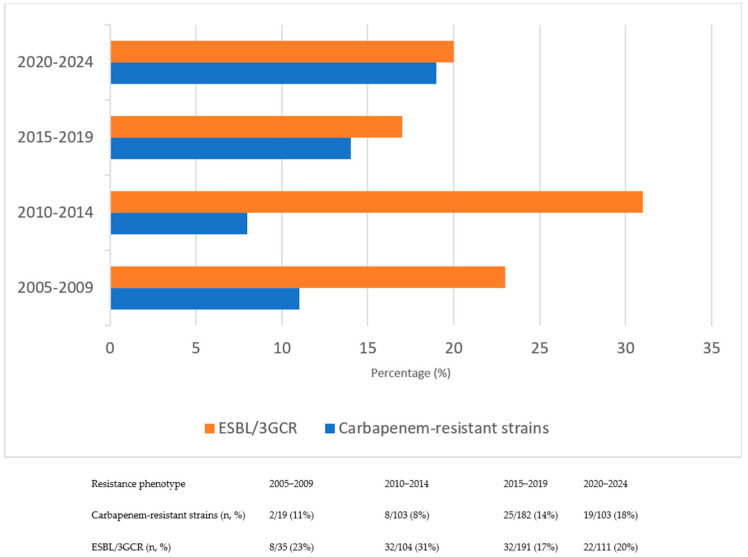
Changes in resistance rates of ESBL/3GCR and carbapenem–resistant strains over time.

The mean time to bacterial growth after hospitalization was 15.3 ± 11 days. As shown in Figure 1, the most frequently isolated pathogen was *Escherichia coli* (53.3%), followed by *Klebsiella* spp. (20.6%) and *Pseudomonas* spp. (7.5%). Among the isolated organisms, 21% exhibited ESBL/3GCR, while carbapenem resistance was identified in 13%. To evaluate resistance trends, the study period was initially divided into four equal intervals to examine temporal changes in antimicrobial resistance patterns. The analysis revealed a progressive increase in carbapenem resistance over time, whereas ESBL/3GCR resistance reached its highest level during the 2010–2014 interval. Temporal trends in antimicrobial resistance patterns across the study period are presented in Figure 2.

Antibiotic use was documented in 405 episodes. Among them, β-lactam antibiotics were administered in 401 episodes, and 386 patients initially received a non-carbapenem β-lactam. Quinolones were used in 49 episodes, 45 of which were given concomitantly with a β-lactam agent. Glycopeptides were added in 78 episodes. Carbapenem use was recorded in 99 episodes: antimicrobial therapy was escalated to a carbapenem in 84 episodes based on clinical deterioration or microbiological results, while 15 episodes were started directly on a carbapenem regimen; one of these also received a glycopeptide. Antifungal therapy was administered in 45 episodes. The median duration of antibiotic treatment was 17 days.

The median duration of fever was 4 days (Q1–3; 2–8). Follow-up blood cultures obtained at 48–72 h after the initiation of empirical antibiotic therapy remained positive in 46 patients. In these control cultures, *Escherichia coli* remained the most common isolate, followed by *Klebsiella* spp. and *Pseudomonas* spp.

In 99 episodes, the primary focus of bacteremia was identified. The median length of hospital stay was 29 days, and 15% of episodes required admission to the ICU. The overall mortality rate was 26.7% (96 out of 362 episodes), with outcomes unknown in 79 cases.

We compared survivors and non-survivors to delineate the differences between the two groups. In comparing the cases, our findings suggest that ESBL/3GCR organisms, and carbapenem-resistant isolates, as well as persistent bacteremia resulting from inadequate source control may be associated with increased mortality. Mortality was also more frequently observed among patients admitted to the ICU. Furthermore, persistence of fever, along with elevated C-reactive protein and procalcitonin levels, was more commonly noted in non-survival, as detailed in Table 2.

## 4. Discussion

Defining the epidemiology of bloodstream infections in patients with hematological malignancies is essential for providing evidence-based guidance on empirical treatment in these high-risk populations. BSIs caused by GNB remain a major cause of morbidity and mortality in patients with hematological malignancies. Our study represents one of the largest single-center analyses addressing this vulnerable population, providing valuable insights into both microbiological characteristics and clinical outcomes. In our cohort, *Escherichia coli* was the most frequently isolated pathogen, followed by *Klebsiella* spp. and *Pseudomonas* spp., reflecting the predominance of *Enterobacterales* as previously reported in similar settings [3,18,19], and a similar pattern has also been documented among oncological patients [2]. Intensive chemotherapy for hematologic malignancies leads to profound myelosuppression and mucosal injury, predisposing patients to Gram-negative bacteremia. Disruption of mucosal barriers is estimated to account for roughly half of all bloodstream infections in patients with cancer [20]. Puerta-Alcalde and colleagues [7] also documented, in their 25-year study of hematopoietic stem cell transplant recipients, a progressive increase in the incidence of Gram-negative bacilli over time.

According to IDSA guidelines for febrile neutropenia in patients with hematologic malignancies, empirical regimens should be tailored according to risk stratification, with a priority on Gram-negative coverage, including *Pseudomonas aeruginosa* in high-risk settings [14]. Although *Pseudomonas* spp. is considered a critical pathogen in febrile neutropenia, its frequency in our cohort was relatively low (7.5%). In contrast, *Enterobacteriaceae* accounted for the vast majority of isolates. A recent multicenter study of *P. aeruginosa* bacteremia in hematologic patients reported high rates of antimicrobial resistance to guideline-recommended agents, leading to frequent inappropriate empirical treatments and increased mortality [21,22]. Together, these insights argue for a targeted empirical approach, employing anti-pseudomonal coverage primarily in patients with specific risk factors, such as prior *Pseudomonas* colonization, prolonged neutropenia, ICU admission, invasive devices, or prior broad-spectrum antibiotic exposure [21,22,23,24,25,26].

These findings suggest that while empirical regimens with antipseudomonal coverage remain recommended in high-risk hematology patients, the actual necessity of routine broad antipseudomonal therapy may need to be reconsidered in centers where the prevalence of *Pseudomonas* bacteremia is low. Overuse of carbapenems or antipseudomonal β-lactams may contribute to further resistance, and tailoring empiric regimens to local epidemiology could provide both effective coverage and improved antimicrobial stewardship.

Antibiotic resistance is one of the most significant global problems, and numerous studies have demonstrated that HM patients are particularly vulnerable [1,4,5,6,10,11,12]. Our analysis showed that 21% of Gram-negative isolates exhibited ESBL/3GCR, with the highest prevalence observed during the 2010–2014 interval. The proportion of ESBL/3GCR among GNB in our study was comparable to that reported in earlier epidemiological investigations, likewise demonstrating stability or a modest decrease over time [25,26,27]. However, this apparent decline may not reflect a true reduction in the frequency of resistance. CLSI revised the minimum inhibitory concentration (MIC) breakpoints for β-lactam antibiotics, including cephalosporins, against *Enterobacterales* in 2010 and 2014 [28]. In our study, CLSI breakpoints were applied for the 2005–2015 period, after which the EUCAST breakpoints (2015–2024) were adopted. These methodological transitions likely contributed to the observed decrease in ESBL prevalence by reclassifying certain isolates as susceptible under updated interpretive criteria, indicating that the decline reflects a methodological rather than an epidemiological change. Given the nearly two-decade study period, changes in diagnostic methods, empirical treatment practices, and susceptibility testing systems over time should also be taken into account when interpreting these trends.

In contrast, carbapenem resistance was detected in 13% of isolates and exhibited a progressive upward trend throughout the study period, consistent with the alarming increase in carbapenem-resistant *Enterobacterales* and non-fermenters described in contemporary European and global data [6,29,30]. This rise can be attributed to intense selective pressures resulting from empirical and prophylactic antibiotic use, particularly the heavy reliance on carbapenems in hematology units [22,25]. Such extensive and prolonged carbapenem exposure may have accelerated the emergence and dissemination of Carbapenemase-producing strains while simultaneously diminishing the relative prevalence of ESBL-producing organisms, a pattern consistent with observations from other tertiary-care hematology centers.

The most used antipseudomonal antibiotics were often inactive against resistant infections, resulting in inadequate therapy for these patients. Therefore, when initiating empirical treatment, institutional and regional surveillance data should be carefully reviewed to guide antibiotic selection. Antibiotic consumption in our cohort was substantial, with β-lactam agents administered to the vast majority of episodes (386 of 405), quinolones to 49, and glycopeptides to 78, while carbapenems were used in nearly one quarter (98 episodes).

We also found that ESBL/3GCR and carbapenem-resistant GNB were detected more frequently in non-survivors. Consistent with our findings, several previous studies in patients with hematologic malignancies and BSIs have reported a strong association between antibiotic resistance among GNB and increased mortality [4,25,26,30,31,32,33]. This may be related to the fact that these organisms are often intrinsically resistant to multiple antibiotic classes and frequently require broader or combination empirical therapy. Delays in initiating effective treatment, particularly in neutropenic or immunocompromised hematology patients, may therefore contribute to poorer outcomes in these infections.

The overall in-hospital mortality rate in our cohort was 26.5%, which is similar to, or in some reports even higher than, that reported in prior studies of BSIs in patients with HM [4,7,8,9,19].

Trecarichi et al. [12] reported that third-generation cephalosporin resistance in *Enterobacteriaceae* bloodstream isolates was associated with higher mortality, with rates increasing from 5% in susceptible cases to 26% in resistant ones. Likewise, Park et al. [4] documented that carbapenem resistance independently increased the risk of death in Gram-negative bacteremia. Evidence from the broader literature further indicates that the presence of carbapenem resistance is associated with increased mortality even in patients without underlying malignancy [14,30].

Comparative analysis showed that mortality in our cohort was influenced by admission to the intensive care unit, reflecting the clinical severity of BSI, a factor previously reported to be associated with mortality in hematological malignancy patients with BSI. Additionally, the presence of persistent fever and ongoing bacteremia appeared to signal inadequate source control, a factor well recognized as a driver of poor outcomes. These features, together with elevated C-reactive protein and procalcitonin levels, were significantly more frequent among non-survivors. Similar associations have been reported in previous studies of bloodstream infections in hematology and critical-care populations, where inadequate source control and delayed eradication of infection were independently linked to higher mortality rates [31,32,33]. Erdem and colleagues reported that Gram-negative bacteremia increased mortality by nearly threefold (OR 2.894; 95% CI 1.437–5.825) and, similar to our findings, observed that approximately one-third of patients presented with a focal infection, while the remaining patients had no identifiable focus [3]. Our findings reinforce these observations and highlight the need for early recognition and aggressive management to improve survival in patients with complicated bacteremia. In this context, recent studies suggest that simple immuno-inflammatory markers, including the neutrophil-to-lymphocyte ratio and platelet dynamics, alongside MDR risk stratification tools, may enhance early prognostic assessment in hematology patients at increased risk for resistant GNB [34]. Although these markers were not available in our dataset, their incorporation into future analyses may further improve early risk assessment in this vulnerable population.

This study has some limitations. It was a single-center investigation, which limits the generalizability of the findings to regions with different epidemiological settings. Although the study spanned a long observation period, some clinical and microbiological data were missing, particularly in earlier years. Leukemia subtypes could not be analyzed separately because chronic leukemia cases were few and subtype information was missing for 38 patients, limiting the ability to perform meaningful subgroup comparisons between acute and chronic leukemias. The absence of standardized clinical severity scores such as SOFA or APACHE II further limits the assessment of illness severity in this cohort. Given the retrospective design and the limited number of outcome events, multivariate modeling could not be performed to identify factors associated with mortality. In addition, the molecular mechanisms underlying resistance in the GNB isolates were not investigated, restricting insight into the genetic determinants of resistance.

In conclusion, *Escherichia coli* remained the predominant isolate in this large cohort of hematology patients, while carbapenem-resistant strains represented an increasing clinical challenge. Carbapenem resistance, ESBL/3GCR resistance, and persistent bacteremia were each associated with higher mortality, underscoring the need for ongoing surveillance, judicious use of broad-spectrum antibiotics, reinforcement of infection control measures, and locally informed empirical therapy and stewardship strategies in this high-risk population. Early recognition of these high-risk features may improve clinical outcomes. Future research should aim to validate these findings in prospective multicenter studies and to better define the temporal dynamics of resistance in hematology populations. Efforts to refine empirical treatment algorithms based on emerging local resistance patterns may further improve outcomes in patients with high-risk GNB.

## Figures and Tables

**Table 1:** Demographic and baseline clinical characteristics of the study population.

	Leukemia *n* (%)	Lymphoma *n* (%)	Others *n* (%)	Total *n* (%)
Sex (male), *n* (%)	208/356 (58.4)	37/59 (62.7)	15/26 (57.7)	260/441 (59)
Age, years (SD)	44.57 (14.9)	52.89 (15.05)	53.22 (18.36)	46.19 (15.47)
Duration of malignancy, years (SD)	1.07 (1.67)	1.04 (1.46)	1.67 (2.22)	1.09 (1.68)
Fever, *n* (%)	287/291 (98.6)	42/46 (91.3)	18/19 (94.7)	347/356 (97.5)
Fever duration (day) median (Q1–3)	4 (2–8)	2 (1–4.25)	4 (1–14)	4 (2–8)
Patients with neutropenia, *n* (%)	272/300 (90.7)	37/47 (78.7)	12/20 (60.0)	321/367 (87.5)
Duration of neutropenia, days (SD)	16.69 (13.56)	6.32 (6.82)	14.42 (21.07)	15.23 (13.8)
Hospitalization (days) median (Q1–3)	28 (22–37)	20 (14.75–26.25)	21.5 (7–43.5)	27 (20–35)
Treatment duration (days) median (Q1–3)	15 (11–24)	10 (5.5–14)	11.5 (5.25–19.25)	14 (10–21.25)
ICU admission, *n* (%)	46/288 (16)	6/45 (13.3)	2/17 (10.5)	54/352 (15.3)
All-cause in-hospital mortality, *n* (%)	78/298 (26.2)	15/46 (32.6)	3/18 (16.7)	96/362 (26.5)
Lab value (min–max)				
White blood cell counts (normal range: 4–10 × 10^3^/ µL)	2385.9 (0–121,000)	4214.7 (0–65,400)	4213.5 (100–27,000)	2721.5 (0–121,000)
Absolute neutrophil count (cells/µL)	552.6 (0–35,690)	1778.9 (0–17,060)	3028.5 (0–25,000)	846.2 (0–35,690)
C-reactive protein (mg/L, normal range: 0–5)	122.7 (0.4–668)	135.7 (3.1–407)	119.7 (0.5–463)	124.2 (0.4–668)
Procalcitonin (µg/L, normal range <5)	10.1 (0–100)	6.5 (0.1–100)	5.9 (0.1–37)	9.3 (0–100)
Time from hospital admission to positive blood culture, days (SD)	15.9 (11.41)	12.11 (7.33)	13.11 (10.28)	15.27 (10.98)
Presence of ESBL/3GCR, *n* (%)	77/356 (21.6)	11/59 (18.6)	6/26 (23.1)	94/441 (21.1)
Presence of carbapenem resistance, *n* (%)	46/333 (13.8)	6/52 (11.5)	2/22 (9.1)	54/407 (13.3)
Persistence of bacteremia In follow-up blood cultures, *n* (%)	38/244 (15.6)	3/33 (9.1)	5/14 (35.7)	46/291 (15.8)

All values were calculated with respect to the number of episodes. ICU: intensive care unit, ESBL/3GCR: Extended-Spectrum β-Lactamase-producing/Third-Generation Cephalosporin-Resistant.

**Table 2:** Comparison of clinical, microbiological, and laboratory characteristics between survivors and non-survivors.

Parameters	Survivors	Non-Survivors	*p* Value	OR
Age meadian (Q1–3)	49.1 (32.5–59.7)	49 (35.5–58)	0.934	
Sex (male), *n* (%)	158/266 (59.4)	53/96 (55.2)	0.547	0.929 (0.757–1.142)
Leukemia, *n* (%)	220/266 (82.7)	78/96 (81.3)	0.433	
Lymphoma, *n* (%)	31/266 (11.7)	15/96 (15.6)		
Other, *n* (%)	15/266 (5.6)	3/96 (3.1)		
Fever, *n* (%)	254/261 (97.3)	90/92 (97.8)	>0.9	1.005 (0.969–1.043)
Intervention, *n* (%)	32/223 (14.3)	14/69 (20.3)	0.258	1.414 (0.802–2.493)
Persistence of bacteremia In follow-up blood cultures, *n* (%)	17/219 (7.8)	29/70 (41.4)	<0.0001	5.337 (3.126–9.111)
ICU admission, *n* (%)	9/257 (3.5)	44/92 (47.8)	<0.0001	13.657 (6.944–26.859)
Presence of *Pseudomonas* spp., *n* (%)	17/266 (6.4)	12/96 (12.5)	0.059	1.956 (0.970–3.943)
Presence of ESBL/3GCR, *n* (%)	53/266 (19.9)	29/96 (30.2)	0.046	1.516 (1.029–2.235)
Presence of carbapenem resistance, *n* (%)	19/254 (7.5)	23/89 (25.8)	<0.0001	3.455 (1.978–6.034)
Duration of malignancy, years, median (Q1–3)	1 (0–1)	1 (0–1)	0.236	
Fever duration (day) median (Q1–3)	3 (2–7)	8 (3.8–15.8)	<0.0001	
Duration of neutropenia, days median (Q1–3)	11 (7–20)	10 (3–28)	0.635	
Time from hospital admission to positive blood culture, days, median (Q1–3)	14 (11–17)	12 (8–20)	0.395	
Hospitalisation (days) median (Q1–3)	26 (20.3–34)	27.5 (19–42)	0.311	
Treatment duration (days) median (Q1–3)	14 (10–20)	15 (6–30)	0.616	
Lab value, median (Q1–3)				
White blood cell counts (normal range: 4–10 × 10^3^/µL)	210 (85–540)	205 (100–1800)	0.084	
C-reactive protein (mg/L, normal range: 0–5)	87 (28.5–172)	137 (62–211)	0.001	
Procalcitonin (µg/L, normal range <5)	0.3 (0.12–1.7)	0.7 (0.2–8.5)	0.046	

All values were calculated with respect to the number of episodes. The analysis comparing survivors and non-survivors was conducted on 362 episodes, as outcome data were missing for 79 episodes. ICU: intensive care unit, ESBL/3GCR: Extended-Spectrum β-Lactamase-producing/Third-Generation Cephalosporin-Resistant.

## Data Availability

The datasets generated during the current study are not publicly available due to the hospital’s data protection policy, but are available from the corresponding author upon reasonable request. Additional Information: Correspondence and requests for materials should be addressed to F.A.
